# COVID-19 Policy Decisions in Manitoba and the Experiences of the Red River Métis: A partnership-based, whole-population linked administrative data study

**DOI:** 10.23889/ijpds.v9i5.2869

**Published:** 2024-09-18

**Authors:** Danielle Saj, Olena Kloss, Francis Chartrand, Oke Ekuma, Carole Taylor, Julianne Sanguins, Alan Katz, Alyson Mahar, Michelle Driedger, Nathan Nickel

**Affiliations:** 1 University of Manitoba; 2 Manitoba Metis Federation; 3 Queen's University

Systematically marginalized populations, like Red River Métis, have been greatly affected by COVID-19. Manitoba’s Indigenous COVID-19 vaccine policy initially delayed prioritization of Métis. Our research team, which included Métis partners, examined the consequences of these decisions (COVID-19 infections, health service use, vaccine uptake) among Métis, and how earlier prioritization could have improved outcomes.

This retrospective cohort study linked data from the Métis Population Database to whole-population COVID testing and vaccination data, and administrative data on health service use. Restricted mean survival time models tested whether vaccination uptake differed between Métis and all other Manitobans (AOM), adjusting for sociodemographic characteristics and comorbidities. A Bayesian model will simulate how prioritization of Métis for vaccination two weeks earlier could have impacted infections.

Cumulative prevalence of COVID-19 infection rates were similar among Métis and AOM until May 2021 when rates became higher among Métis. Between May and December 2021, rates of first vaccination were lower among Metis than AOM, as were second vaccination rates between July and November 2021. Métis were more likely than AOM to be hospitalized due to Covid-19 between March and August 2021 and visit physicians for COVID related reasons from October 2020 to Feb 2021 and November 2021 to March 2022. Our analyses simulated what would have occurred had Métis been prioritized for vaccination two weeks before AOM, alongside other Indigenous peoples.

Understanding the experiences of Métis relative to AOM is critical to identifying public health strategies which close gaps in vaccine uptake and infections.

